# List of Licentiates

**Published:** 1877-11

**Authors:** 


Official List of Licentiates of the Illinois State Board of Health who have qualified since the last announcement made in this Journal:

Frederick Seymour, Cincinnati College of Medicine and Surgery..........Feb. 18th, 1856
Daniel Lord, Chicago Medical College..........March 13th, 1873
Charles N. Dorion, Hahnemann Medical College, Chicago..........March 23d, 1869
Isadore Cohen, the University of Vienna, Austria.......... July 21st, 1873
C. A. Helmuth, the University of Berlin, Prussia..........1844
John Henry Byrne, Rush Medical College..........Feb. 17th, 1874
G. L. Henderson, Chicago Medieal College..........March 9th, 1868
E. F. Ingals, Rush Medical College..........Feb. 1st, 1871
William Goetz. University of Tubingen, Wurtenburg, Germany..........May, 1842
D. R. Brower, Med. Department, Georgetown College, Georgetown, D.C...March 3d, 1864
Jno. R. Buchan, Long Island Hospital Medical College, Brooklyn, N. Y...June 26th, 1872
N. P. Pearson, University of Copenhagen, Copenhagen..........June 20th, 853
George W. Hilton, Hahnemann Medical College, Chicago..........Feb. 22d, 1877
Charles J. Simons, Albany Medical College, Albany, N. Y..........Dec. 24th, 1876
William H. Woodbury, Hahnemann Medical College, Chicago..........March 1st, 1866
R. H. Bingham, Castleton Medical College, Castleton, Vt..........Nov. 21st, 1849
D. A. K. Steele, Chicago Medical College, Chicago..........March 13th, 1873
Leonard St. John, Royal College of Surgeons, London, Eng..........Nov. 20th, 1873
A. H. Tagert, The University of Vermont, Burlington, Vt..........June 9th, 1866
E. M. P. Ludlam, Hahnemann Medical College..........Feb. 26th, 1861
A. E. Small, Medical Department Pennsylvania College, Philadelphia, Pa...March, 1842
George W. Goodner, Chicago Medical College, Chicago..........April 10th, 1869
S. D. Jacobson, The University of Copenhagen, Denmark..........June 20th, 1862
F. L. Breidenstein, The University of France, Strasburg..........Sept. 10th, 1841
W. K. Evarson, Eclectic Medical College of Pennsylvania.
L. W. Case, Rush Medical College, Chicago..........Feb. 2d, 1870
John Schuller, University of Giesen-Giesen, Hesse Darmstadt..........Dec. 6th, 1854
Matthew O. Jones, Medical Department University of Pennsylvania, Pa...March 8th, 1850
E. W. Edwards, Washington College, Baltimore, Md..........Feb. 20th, 1846
Frank W. Edwards, Rush Medical College, Chicago..........Feb. 22d, 1876
A. W. Gray, Chicago Medical College, Chicago..........March 4th, 1868
J. A. St. John, Chicago Medical College, Chicago..........March 13th. 1873
F. B. Ives, Rush Medical College, Chicago..........February, 1850
Geo. M. Emmerick, Chicago Medical College..........March 13th, 1873
Charles E. Davis, University of Michigan..........March 1st 1874
Eugene A. Mullan, Jefferson Medical College..........March 11th, 1874
C. C. Higgins, University of Iowa, Keokuk..........June 25th, 1863

Allen W. Hagenbuch, Rush Medical College.....Feb. 15th, 1876
Peter J. Rowan, University of Toronto, Canada.....June 8th, 1870
A. S. MacLennan, Royal Col. of Physicians and Surg., Kingston, Canada..Mar. 20th, '73
S. Saur, Georgetown College, D. C.....March 15th, 1855
A. S. Munnsell, Chicago Medical College.....March 14th, 1871
A. L. Freund, Bennett Medical College.....Feb. 21st, 1877
John H. Hollister, Berkshire Medical College.....Nov. 7th, 1847
George W. Gurnea, Rush Medical College.....Feb. 21st, 1877
Temple S. Hoyne, Bellevue Hospital Medical College.....March, 1865
John B. Braun, University Erlangen, Bavaria,.....June 14th, 1848
C. P. Simon, University of Leipsic, Saxony.....Jan, 2d, 1864
James Lawless, Rush Medical College.....Feb. 21st, 1877
A. R. Jackson, Medical Department of Pennsylvania College.....March 7th, 1848
T. Hoffman, Rush Medical College.
G. A. H. Sienanke, Chicago Medical College.....March 20th, 1877
J. F. Schafer, Rush Medical College.....Feb. 19th, 1873
J. M. Hall, College of Physicians and Surgeons, New York City.....March 3d, 1874
F. Cehavett, Bennett Medical College.....Feb. 27th, 1869
M. H. Luken, Rush Medical College.....Feb. 19th, 1873
W. J. Johnson, Chicago Medical College.....March 4th, 1868
T. C. Duncan, Hahnemann Medical College, Chicago.....March 1st, 1866
Annie E. Bailey, Hahnemann Medical College, Chicago.....Feb. 11th, 1875
R. Tilley. Chicago Medical College.....March 21st, 1871
J. A. Mead, Rush Medical College.....Feb. 15th, 1876
T. S. Bidwell, Western Reserve College, Ohio.....Feb. 24th, 1863
N. L. Hurlbut, Rush Medical College.....1852
Charles G. Smith, University of Pennsylvania.....March, 1851
Helen J. Underwood, Female Medical College, New York City.....March 21st, 1872
L. Pratt, Pennsylvania Homœopathic Medical College.....March 1st, 1852
C. C. Spry, Chicago Medical College.....March 4th, 1874
J R. Kippax, Hahnemann Medical College, Chicago.....March 1st, 1869
Marcus Swain, Dartmouth Medical College, N. H.....Oct. 28th, 1833
T. Wild, Rush Medical College.....Jan. 25th, 1865
N. W. Abbott, Berkshire Medical College.....November, 1844
S. J. Jones, Medical Department University of Pennsylvania.....March 15th, 1860
R. C. Hamill, Licentiate Ohio Med. Col. 1838; Rush Medical College.....Feb. 25th, 1861
H. M. Lyman, College of Physicians and Surgeons, N. Y.....March 14th, 1861
H. Moer, Eclectic Medical College, Cincinnati.....May 28th, 1864
Frank H. Davis, Chicago Medical College.....March 14th, 1871
G. Hessert, University of Wurzburg, Germany.....Aug. 14th, 1858
H. Kirschstein, University of Breslau, Germany.....June, 1862
Jacob Dal, Hahnemann Medical College.....March, 1872
H. P. Merriman, Chicago Medical College.....March 9th, 1865
S. W. Austin, Albany Medical College, Albany, N. Y.....Dec. 28th, 1854
Wm. J. Hawkes, Homœopathic Medical College, Philadelphia.....March 1st, 1867
R. E. Ulrich, University of Wurzburg, Germany.....July 7th. 1871
Lizzie Dyson, Female Medical College of Pennsylvania.....March 15th, 1864
Thomas Bevan, Medical College of Ohio.....March 4th, 1851
A. N. Sheffner, Bennett Medical College, Chicago.....May 21st, 1875
Jacob S Kauffman. Rush Medical College.....Feb. 16th, 1875
George H. Hall, Hahnemann Medical College, Chicago,.....February, 1864
Henry H. Sloan, Chicago Medical College.....March 20th, 1869
L. Bedford, Homœopathic Medical College of Pennsylvania.....Feb. 24th, 1865
C. H. Von Tagen, Homœopathic Medical College, Pennsylvania.....March 2d, 1858
J. A. Lane McGill College, Montreal, Canada.....March 28th, 1877
R. N. Token, Bellevue Hospital. Medical College, New York City.....March 1st, 1875
J. A. McWilliams, Chicago Medical College.....March 1st, 1876
James E. Gross, Hahnemann Medical College, Philadelphia.....March, 1850

N. S. Davis, Fairfield Medical College........ ......................Jan. 31st, 1837
Wm. H. Morgan, Rush Medical College.................... ........Feb. 17th, 1874
J. A. Freeman, American Medical College, Cincinnati. Ohio.................Feb 3d. 1855
J. V. Bachelle, Rush Medical College...................... ................Jan. 25th, 186[illegible]
E. H. Horsey, Queen's University, Kingston, Canada..........................April 15th, 1869
William A. Crocker, Medical Department University of New York ......March 15th, 1876
Peter S. Macdonald, Rush Medical College.................... . ................1864
Arthur B. Hosmer, Chicago Medical College.......................... ...................1875
William S. Johnson, Hahnemann Medical College, Chicago..........................1868
Henry Tomboeken, Rush Medical College...........................................1866
James P. Mills, Hahnemann Medical College, Philadelphia..................... ..........1874
Pierre Marchand, University of Vermont, Burlington, Vt, ...........................1862
Luther C. Bean, Woodstock Medical College, Woodstock, Vt.,................ ......1849
Andrew J. Baxter, Ohio Medical Colleg ,.....................................March 4, 1861
George E. Shipman, College of Physicians and Surgeons, New York City....Sept. 2, 1843
Elihu G. Cook. Western Homœpathic College of Cleveland, Ohio.... .......Feb. 27, 1863
A. B. Wescott, Eclectic Medical Institute, Cincinnati, Ohio,...................Feb. 15, 1857
Louise M. Dawson. Woman's Hospital Medical College, Chicago... .........Feb. 27, 1877
Albert G. Beebe, Bellevue Hospital Medical College, New York ... .........March 1, 1870
Juliet Caldwell, Homœpathic Medical College, University of Michigan....March 28, 1877
Edmund C. Rogers, Albany Medical College .................................January 25, 1853
James Nevins Hyde, Medical Department University of Pennsylvania....March 13, 1863
Fred. A. Beck, Chicago Medical College,..... ............. .....................March 20, 1877
Edward B. Taylor, Bennett Eclectic Medical College, Chicago.............. Feb. 21, 1877
Alfred G. Schloesser, Rush Medical College.......................................Feb. 1, 1871
William R. McLaren, Hahnemann Medical College, Chicago.....................Feb. 20, 1871
Samuel P. Hedges, Hahnemann Medical College, Chicago.........................March, 1867
A. B. Newkirk, Starling Medical College, Columbus, Ohio................ ...March 1, 1849
Robert B[illegible]sch. University of Rostock, Germany..................................Nov. 2, 1864
Mary A. Ries, Bennett Eclectic Medical College, Chicago........................Feb. 22, 1877
Ferdinand C. Hotz, University of Heidelberg, Germany..............................Dec. 16. 1865
Nelson H. Church, Rush Medical College, Chicago....................................Feb. 3, 1869
Eugene Marguerat, University of New York, New York City ..................March, 1859
Christopher C. P. Silva, Funchal College, Madeira Isl'd, Portugal...........October, 1861
George O. Rutledge, Chicago Medical College...................................March 20, 1877
William P. Dunne, Rush Medical College..........................................Jan. 25, 1867
Julia H. Smith, Chicago Homœopathic Medical College...........................March 5, 1877
George A. Hall, Homœpathic College, Philadelphia.. .......................... March 1, 1856
Raymond L. Leonard. Rush Medical College.......... ..........................Feb. 17, 1872
S. C. Pitcher, Jefferson Medical College, Philadelphia................ ...........March 10, 1864
Fred. F. Laws, Chicago Medical College..... .....................................March 10, 1874
Hans Jacob G. Koren, Collegium Fredericianæ Regiæ Academicum Universitatis, City of Christiania, Norway...................................June 7, 1869
F. R. Mergler, University of Wurzburg, Germany................. ...........Feb. 15, 1840
Fred. Schubart, University of Heidelberg, Germany.............................Nov. 24, 1851
Harry Brown, McGill College, Montreal, Canada...............................March 18, 1873
Patrick M. O'Connell, Missouri Medical College, St. Louis......................March 2, 1877
James H. Etheridge, Rush Medical College................ .. ...................Feb. 4, 1869
William W. Winter Eclectic Medical Institute, Cincinnati, Ohio.............May 17, 1859
Edward H. Webster, Chicago Medical College............... ...............March 20, 1877
B. P. Reynolds, Eclectic Medical College of Pennsylvania, Philadelphia......March, 1863
H. C. Jessen, Hahnemann Medical College of Missouri, St. Louis............March 1, 1874
Geo. W. Williams, Chicago Medical College......................................March 13, 1873
George Kellogg, Buffalo Medical College, Buffalo, N. Y.............................Feb. 25, 1854
John W. Tope, Rush Medical College..............................................Feb. 2, 1870
Edmund Andrews, Medical Department University of Michigan..............April 6, 1852
Charles L. Rutter, Chicago Medical College.......................................March 4, 1868
Sigismund D. Neve, Hahnemann Medical College, Chicago.......................Feb. 22, 187

Benjamin E. Dailey, Georgetown College, D. C. .......................... March 10, 1874
E. G. H. Miessler, Hahnemann Medical College, Chicago .......................... Feb., 1873
Jacob Breen, Georgetown Medical College, D. C. .......................... March 9, 1871
Helen A. Heath, Chicago Homœpathic Medical College .......................... March 5, 1877
David S. Smith, Jefferson Medical College, Philadelphia .......................... March 14, 1836
Mary H. Thompson, Chicago Medical College .......................... March 22, 1870
Henry N. Small, Hahnemann Medical College, Chicago .......................... March 1, 1866
David B. Trimble, Jefferson Medical Coll ge, Philadelphia .......................... March 12, 1837
Chas. H. Lovewell, Universiay of Michigan .......................... March 29, 1871
F. L. Wadsworth, Ru-h Medical College .......................... Feb. 3, 1869
Albert E. Hoadley, Chicago Medical College .......................... March 12, 1872
Henry Banga, University of Basle, Switzerland .......................... Sep. 16, 1871
Oliver T. Shenick, Rush Medical College .......................... Feb. 21, 1877
Xenophon C apman, Chicago Medical College .......................... March 10, 1874
C. J. Lewis, Rush Medical College .......................... Jan. 25, 1865
Christian Fenger, University of Copenhagen, Denmark .......................... Jan. 1867
Ephraim Ingals, Rush Medical College .......................... Feb. 14, 1847
Isidor Sax, Hahnemann Medical College, Chicago .......................... Feb. 24, 1877
Martin Phillips, Victoria College, Toronto, Canada .......................... April 4, 1862
T. Davis Fitch, Rush Medical College .......................... Feb., 1854
William Reinhold, Rush Medical College .......................... Feb. 5, 1868
Isaac N. Lilly, University of Louisville, Ky .......................... Feb., 1864
F. E. Sherman, Rush Medical College .......................... Feb. 17, 1873
W. C. Westerfield, St. Louis Medical College, Missouri .......................... March 2, 1859
Thomas A. Lilly, Kentucky School of Medicine, Louisville .......................... March, 1864
J. Ramsey Hood, Jefferson Medical College, Philadelphia .......................... March 10, 1866
Henry Geiger, University of Heidelberg, Germany .......................... Dec. 1861
James W. Hutchinson, Chicago Medical College .......................... March 3, 1867
C. W. Burrill, Chicago Medical College .......................... March 12, 1872
A. B. Clark, Chicago Medical College .......................... March 21, 1876
Henry T. Byford, Chicago Medical College .......................... March 13, 1873
Roswell Park, Chicago Medical College .......................... March 21, 1876
Geo. Keating Dyas, Chicago Medical College .......................... March 21, 1869
Samuel H. Bottomley, Medical Department Lind University .......................... March 3, 1863
Alex. Steel, University City of New York .......................... March 5, 1866
Louis Braun, R. M. College .......................... Feb. 15, 1876
A. W. Smith, Eclectic Medical Institute, Cincinnati, Ohio .......................... May 21, 1872
Patrick Henry McElroy, R. M. College .......................... Feb. 1, 1871
Rudolph Seiffert, University of Vienna, Austria .......................... Feb. 13, 1852
Hosmer A. Johnson, R. M. College .......................... Feb., 1852
Harlan P. Cole, N. Y. Hom r Medical College .......................... March 3, 1872
" Chicago Medical College .......................... March 14, 1871
Henry Hooper, Medical Department Harvard University .......................... June, 1869

